# Utilization possibilities of steel slag as backfill material in coastal structures

**DOI:** 10.1038/s41598-023-31156-z

**Published:** 2023-03-15

**Authors:** Gulsen Tozsin, Fatih Yonar, Onuralp Yucel, Atilla Dikbas

**Affiliations:** 1 Department of Metallurgical and Materials Engineering, Ataturk University, 25240 Erzurum, Turkey; 2 Department of Civil Engineering, Çanakkale Onsekiz Mart University, 17020 Çanakkale, Turkey; 3 Department of Metallurgical and Materials Engineering, Istanbul Technical University, 34469 Istanbul, Turkey; 4 ARI Teknokent, Istanbul Technical University, 34467 Istanbul, Turkey

**Keywords:** Environmental sciences, Engineering, Materials science

## Abstract

The aim of this study is to investigate the utilization possibilities of steel slags, basic oxygen furnace (BOF) and electric arc furnace (EAF) slags, as backfill material in coastal structures. Within the scope of the study, physical, mechanical and chemical properties of the steel slags were investigated and their potential to create environmental risks were evaluated. The results showed that soundness loss and filler content ratio were below the limit values for steel slags to be used as backfill material. It was determined that the density, porosity, water absorption and Los Angeles abrasion ratios of steel slags were generally higher than natural aggregates. In order to reach the California Bearing Ratio (CBR) limit (> 25%), the maximum particle size of the steel slag was reduced to 25 mm. In this particle size, CBR of the slag samples generally gave better results compared to the natural aggregate (38%), except for Kardemir and Asil samples. In addition, the concentration values of heavy metals (Cu, Cd, Cr, Pb, Ni, Zn, Hg and As) were below the limit values specified in the regulation. It is suggested that EAF slags should be aged for at least 6 months and BOF slags for at least 24 months in open air conditions before being used as backfill material in coastal structures after the maximum particle size is reduced to 25 mm.

## Introduction

Considerable quantities of slags are generated as by-products from steel industries^1^. Steel slags are obtained from the separation of impurities in molten steel during the reduction of liquid crude iron to liquid crude steel in basic oxygen furnaces (BOF) or during the reduction of scrap to liquid crude steel in electric arc furnaces (EAF)^2,3^. According to the statistics of 2020, Turkey is the 7th largest steel producer in the world and the largest steel producer in Europe with its steel production of 35.8 million tons^4^. During the production of 1-ton liquid crude steel in BOF and EAF, steel slag occurs at a ratio of 12–15%^5–7^. The steel slag generally contains CaO, MgO, SiO_2_, Al_2_O_3_, Fe_2_O_3_, MnO and P_2_O_5_ compounds in its structure^8–10^.

For steel slag to be used in construction applications, free CaO and MgO compounds, which cause expansion, must be hydrated, in other words, slag must be aged. In the first stage during the aging process of the slag, CaO and MgO are converted into Ca(OH)_2_ and Mg(OH)_2_, respectively, through hydration. When the aging process is kept sufficiently long, Ca(OH)_2_ and Mg(OH)_2_ turn into CaCO_3_ and MgCO_3_, respectively, with the influence of CO_2_^11–15^. In studies conducted by Aiban^16^ and Sofilic et al.^17^, heavy metal concentrations of EAF slags were investigated. The results obtained were compared with the limits of the United States Environmental Protection Agency (USEPA) and DIN 38,414-S4, respectively and it was stated that EAF slags are non-hazardous by-products. In the Taiwan South-Star Project carried out between 1996 and 2012, 4.55 million tons of BOF slag was used as coastal structures backfill material for an area of 113 hectares. Once the construction was completed, samples were collected from 12 different points, 1 m below sea level, 5–7 m deep and 1 m above the bottom and changes in heavy metal concentrations were observed. In all measurements, Cd < 0.004 mg/L, Pb < 0.001 mg/L, Hg < 0.001 mg/L, Cu < 0.004 mg/L, Zn < 0.008 mg/L and Cr < 0.0004 mg/L were determined^18^. 692 thousand m^3^ steel slag was used as the base material of the quay wall in the expansion work in Hiroshima Port, between 1997 and 1998. 600 thousand m^3^ steel slag was used in the artificial island of Oshima in Japan. Between 2013 and 2015, China Steel Company used BOF slag as aggregate in concrete blocks it produced and these blocks were placed on the stream bank as an erosion-preventing fortification unit in the Kezi Port Emergency Drainage Project. Between 2015 and 2016, China Steel Company produced 6 tetrapods by using BOF slag as aggregate and these tetrapods were placed on the quay. Chubu Centrair International Airport is an example of sea-filled airports in Japan. In the construction of this airport, 320 thousand tons of steel slag was used as the coastal structures backfill material. At Fukuyama Port in Hiroshima, Japan, 250 thousand m^3^ steel slag was used as backfill material in the arrangement made between 2006 and 2007^18^. In the study conducted by Tasalloti et al.^19^, steel slag and coal-washing waste were used together in the extension of Brisbane and Kembla Ports in Australia. The mixed material was compacted as backfill material. Although the mixtures had sufficient bearing capacity and shearing force, 5–6.3% expansion was observed due to the hydration of CaO and MgO in the steel slag. Steel slags are either kept in stock areas or disposed of in sanitary landfills. The storage of steel slags narrows the service areas and their disposal causes a decrease in usable natural spaces as well as increasing the operating cost of the steel producer^13^. Reuse of these materials are essential for conserving mineral resources and protecting the environment^9,20^. Provided that there is no liquefaction or consolidation, soil or granular materials can be used as backfill materials for coastal structures. For this purpose, atterberg limits, maximum dry density, CBR, swelling ratio, soundness, and filler content of backfill materials are investigated according to specifications^21,22^. In this study, possibilities of the utilization of steel slags as backfill material in coastal structures and possible environmental risks associated with these slags were investigated.

## Materials and methods

### Steel slag samples

Steel slags collected from the companies producing with BOF and EAF and the natural aggregate sample used for comparison were examined. Companies where the samples were collected, and slag types are given in Table 1. Material, Construction, Control and Maintenance/Repair Technical Specification for Coastal Structures and Ports^21^ and Technical Specification for Soil Works^22^ are used as the directive documents for physical and mechanical characterization of steel slag as coastal backfill material.

**Table 1:** Companies where the samples were collected and slag types.

Slag types	Companies
BOF Slag	Erdemir (Eregli Iron and Steel Works Company)
	Isdemir (Iskenderun Iron and Steel Works Company)
	Kardemir (Karabuk Iron and Steel Industry and Trade Company)
EAF slag	Asil Steel Industry and Trade Company
	Cebitas Iron and Steel Industry Company
	Cemtas Steel Machinery Industry and Trade Company
	Izmir Iron and Steel Industry Company
	Ozkan Iron and Steel Industry Company
	Yazici Iron and Steel Industry and Trade Company
	Yesilyurt Iron and Steel Industry Company
Natural aggregate	Alyans Construction and Trade Company

### Characterization of the steel slags

According to Material, Construction, Control and Maintenance/Repair Technical Specification for Coastal Structures and Ports^21^, the backfill material can be constructed by using one the following materials: (i) borrowed material, (ii) granular aggregate that is not categorized as quarry waste or (iii) surplus categorized granular aggregate from the quarry. Since the steel slag is a granular material, application procedures of granular materials are investigated for the utilization possibilities of steel slag as backfill material in coastal structures. The application procedures, physical and mechanical properties of granular fill materials are defined in TSSW^22^. In the direction of this specification, particle size distribution and filler content ratio were determined according to ASTM C136 and ASTM C117, respectively. The soundness test of aggregates was carried out according to the ASTM C88 to measure the durability of aggregates against freezing and thawing as a result of prolonged exposure to weather conditions.

The liquid limit and plastic limit were determined according to ASTM D4318. The plasticity index value was calculated with the difference between these two values. Density, porosity and water absorption ratios of steel slags were determined according to ASTM C127 (for coarse aggregates) and ASTM C128 (for fine aggregates). Los Angeles (LA) abrasion of the materials was determined within the scope of ASTM C131. Maximum dry unit weight (γ_d,max_) values and optimum moisture content (w_opt_) of steel slags and natural aggregate were defined with standard proctor test according to ASTM D698. California Bearing Ratio (CBR) and swelling ratio were determined according to ASTM D1883. Expansion potential of steel slag was measured according to ASTM D4792, and the results were compared with the limit specified in the PennDOT 408/2020 specifications. The chemical compositions of the slag samples were determined with XRF (Philips PW 1400). Heavy metals in the steel slag were detected with ICP-MS (Agilent 7800).

## Results and discussion

### Physical properties of steel slags

Particle size, soundness of aggregate, filler content, density, porosity, water absorption and LA abrasion, the important parameters that determine the performance of the granular layer, are given in Table 2. The maximum particle size of the steel slags was between 37.5 and 75 mm. The maximum particle size of the natural aggregate was determined as 50 mm. These values were below the limit value (100 mm) specified in TSSW^22^.

**Table 2:** Physical properties of steel slag samples.

Slag types	Companies	Maximum particle size	Soundness (MgSO_4_)	Filler content	Density	Porosity	Water absorp	LA abrasion
		(mm)	(% loss)	(%)	(kg/m^3^)	(%)	(%)	(% loss)
BOF slag	Erdemir	50	2.6	3.0	3.080	9.5	3.1	22
	Isdemir	50	1.1	4.6	3.190	10.2	3.2	21
	Kardemir	50	1.0	0.7	3.140	5.8	1.9	19
EAF slag	Asil	37.5	0.7	0.0	3.080	6.6	2.1	22
	Cebitas	75	0.5	0.0	3.290	6.5	2.0	19
	Cemtas	75	0.5	0.3	3.160	12.5	3.9	33
	Izmir	75	1.3	0.4	2.760	11.8	4.3	30
	Ozkan	75	0.9	0.4	2.830	9.1	3.2	23
	Yazici	75	0.6	1.2	2.920	9.5	3.3	28
	Yesilyurt	50	1.6	1.2	2.930	7.9	2.7	25
Natural aggr	Alyans	50	1.5	3.6	2.860	3.1	0.4	19

The soundness test evaluates the resistance of aggregate to degradation from freeze–thaw cycles^23^. The high soundness loss indicates that the aggregate has a high risk of degradation under the effect of freezing and thawing. This subsequent degradation may cause additional stresses in the structures above the granular layer^24^. Therefore, the soundness loss is required to be ≤ 18% according to TSSW^22^. The soundness loss varied between 0.5 and 2.6% in steel slags and were well below the limit value (≤ 18%). (Table 2). The lowest soundness loss was observed in Cebitas (0.5%) and Cemtas (0.5%) samples, while the highest soundness loss was observed in Erdemir sample (2.6%). The soundness loss of natural aggregate sample was determined as 1.5%. In order for a material to be used as granular fill material, the filler content ratio (< 75 µm) is required to be less than 25%^22^. It was observed that the filler content of steel slags varied between 0 and 4.6% and remained well below the limit value. The filler content ratio of natural aggregate was 3.6%. Except for the Isdemir sample (4.6%), the filler content ratio of all slag samples was lower than the natural aggregate. Excessive filler content prevents particle interlocking in the aggregate matrix, reducing the bearing ratio and may cause deformations^25^. The liquid limit and the plasticity index of the material should be ≤ 35% and ≤ 15%, respectively, for materials with plastic properties^22^. Materials such as steel slag and natural aggregate that plastic limit or liquid limit value cannot be observed are defined as nonplastic^26^. Plastic-behaving layers show high humidity sensitivity and show a significant decrease in shear strength when exposed to water, unlike layers containing nonplastic grains^25,27,28^. It is evaluated that the steel slag, which is used as a backfill material in coastal structures, will not cause any problems if it encounters precipitation or sea water due to its non-plastic property.

The density of steel slags was very close to or generally higher than the density of the natural aggregate sample (2.860 g/cm^3^) (Table 2). This was due to the high unit weight of the metal oxides in the steel slag. Only Izmir (2.760 g/cm^3^) and Ozkan (2.830 g/cm^3^) samples had lower densities than natural aggregate. High density compared to natural aggregate may increase the transportation cost of steel slag to be used as backfill material. Porosity and water absorption ratios of steel slags were higher than natural aggregate. While the porosity ratio of the natural aggregate was 3.1%, the porosity ratios of BOF slags varied between 5.8 and 10.2% and EAF slags varied between 6.5 and 12.5%. Similarly, while the water absorption ratio of natural aggregate was 0.4%, the water absorption ratios of BOF varied between 1.9 and 3.2% and EAF slags ranged from 2.0 to 4.3%. (Table 2). With the rapid cooling of the steel slag in liquid state, the gases cannot escape and a porous structure is formed^13^. Although the porosity and water absorption ratios of the steel slags were high, it was seen that they were also resistant to weathering. Aggregates with a porous structure were expected to be less resistant to weathering. However, it had been observed that high water absorption did not directly reduce the weathering resistance. Weathering resistance of steel slags appears to be related to the pore size distribution rather than the total porosity. The pore size distribution, which becomes saturated when the aggregate is wet and cannot provide rapid drainage during drying, causes the aggregate to be more affected by weathering effects^29^.

**Figure 1:**
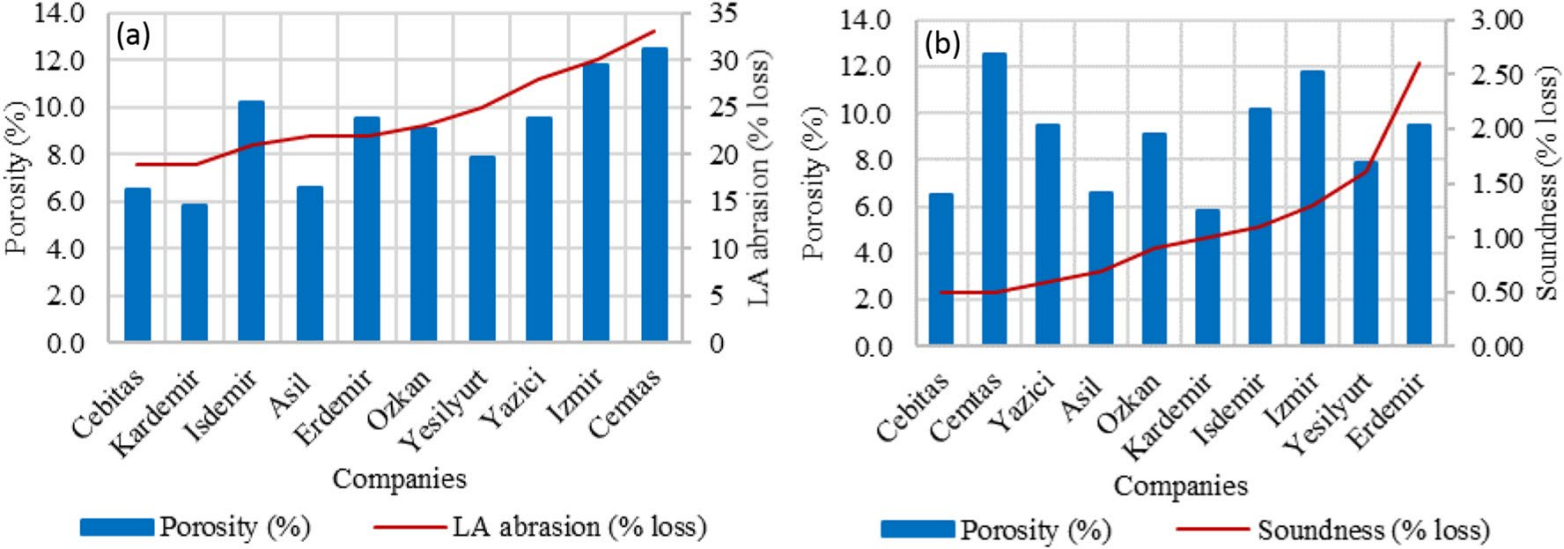
Comparison of Porosity and LA abrasion (%loss) (**a**); Porosity and soundness (% loss) (**b**).

LA abrasion ratios of the steel slags were higher than the natural aggregate sample (19%) (Table 2). Although it is a hard material, gases trapped during rapid cooling are the main cause of the brittle structure of steel slag^30,31^. LA abrasion loss of the Kardemir sample with the lowest porosity ratio (5.8%) was 19%. LA abrasion loss of the Cemtas sample with the highest porosity ratio (12.5%) was 33%. (Table 2). The relationship between porosity-LA abrasion and porosity-soundness loss of steel slags are given in Fig. 1a,b, respectively. As the porosity of the slags increased, the LA abrasion loss increased accordingly (Fig. 1a). Although steel slags had a porous structure, their soundness loss were low (Fig. 1b). While the soundness loss of the Cemtas sample with the highest porosity ratio (12.5%) was 0.5%, the soundness loss of the Cebitas sample with 6.5% porosity was also 0.5%. Under normal conditions, when the porosity increases, the soundness loss is expected to increase^29^. However, it is seen that the increase in the soundness loss of the steel slag samples does not depend on the porosity (Fig. 1b). Lower soundness loss of slag compared to natural aggregate sample indicates the high strength of the slag. However high LA abrasion originates the brittle structure of the steel slag due to the pores formed during rapid cooling^13^.

**Figure 2:**
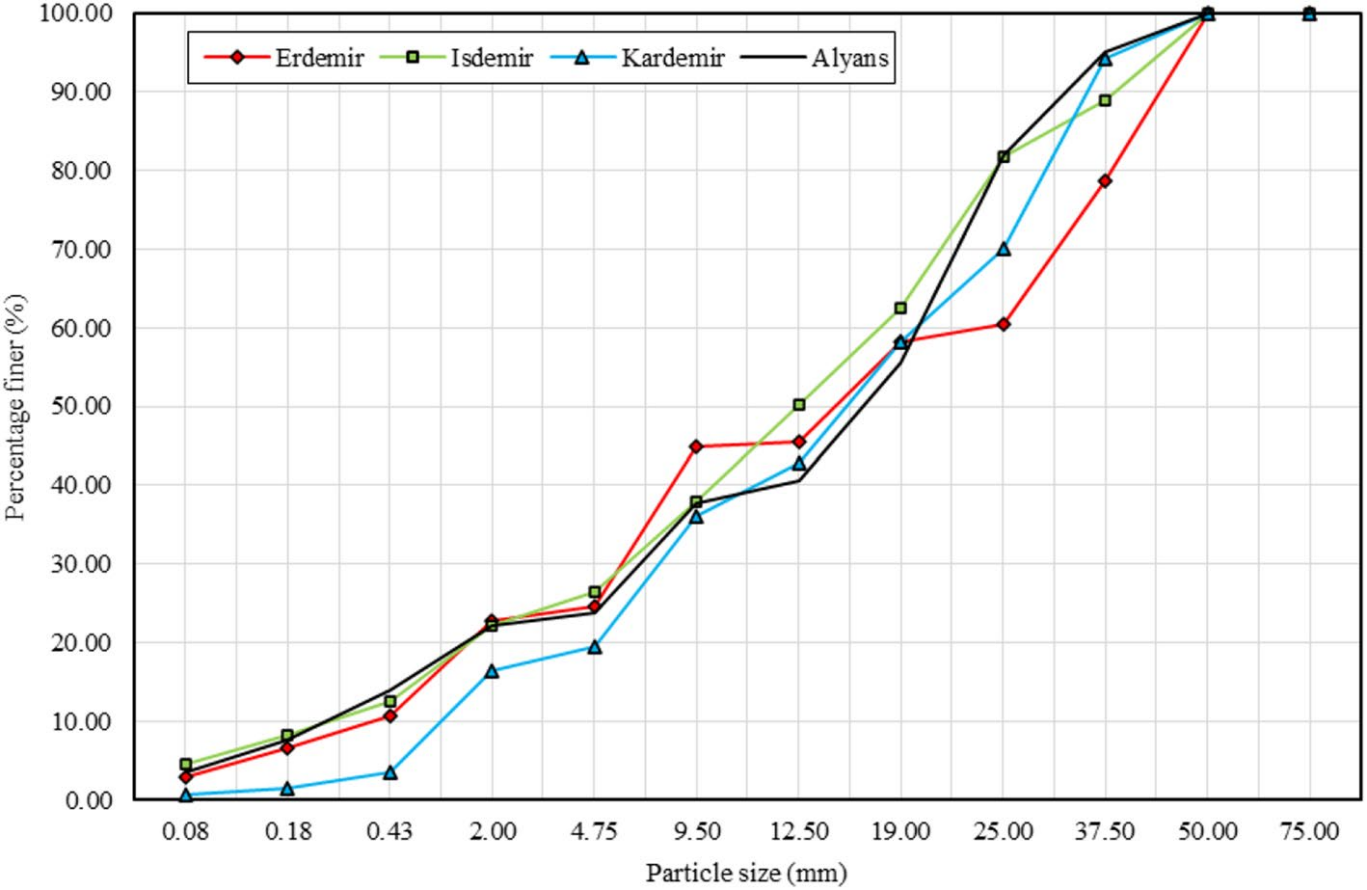
PSD curves of BOF slag samples and natural aggregate.

**Figure 3:**
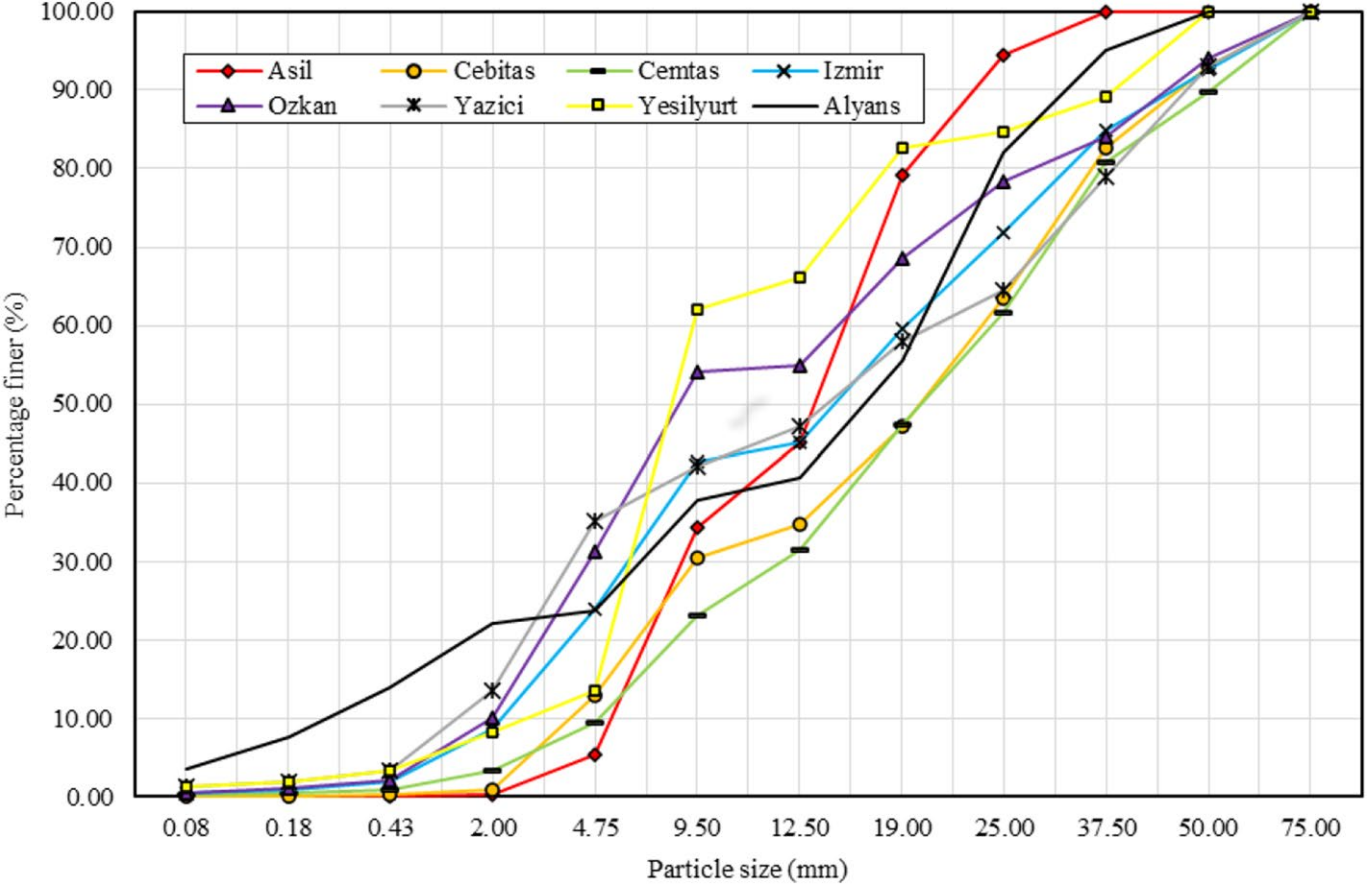
PSD curves of EAF slag samples and natural aggregate.

Particle size distribution (PSD) affects the permeability, erosion susceptibility, frost susceptibility, shear strength, resilient modulus and permanent deformation of materials^32,33^. Dense graded aggregate is defined as a material uniformly graded from coarse to fine, with a distribution of sizes and sufficient mineral filler (< 75 µm) to yield a compacted aggregate having a minimum void space^34^. When the PSD curves in Figs. 2 and 3 are examined, it is seen that the steel slags do not have a dense gradation.

The fine material (< 4.75 mm) ratio of the natural aggregate sample (Alyans) was 23.73% (Figs. 2 and 3). When the mean values were calculated, it was seen that the average fine material (< 4.75 mm) ratios of the BOF (Fig. 2) and EAF (Fig. 3) slag samples were 23.56% and 18.76%, respectively. Compared with the natural aggregate sample, the BOF slag samples show similar granulometry to the natural aggregate, while the EAF slag samples have less fine particles.

### Mechanical properties of steel slags

Maximum dry unit weight (γ_d,max_) and optimum moisture content (w_opt_) determined by applying standard Proctor tests, and CBR (wet) and swelling ratio determined by applying standard CBR tests are given in Table 3.

**Table 3:** Mechanical properties of steel slag samples and natural aggregate.

Slag types	Companies	γ_d,max_ (kN/m^3^)	w_opt_ (%)	CBR, wet (%)	Swelling ratio (%)
BOF slag	Erdemir	15.098	16.86	24	0.330
	Isdemir	17.217	12.40	36	0.477
	Kardemir	21.278	9.34	40	1.850
EAF slag	Asil	16.687	5.14	17	-0.001
	Cebitas	17.305	4.40	28	0.036
	Cemtas	20.660	5.71	28	0.058
	Izmir	18.892	5.45	10	0.037
	Ozkan	19.512	4.67	13	0.056
	Yazici	18.364	8.09	28	0.015
	Yesilyurt	18.806	6.33	26	0.000
Natural aggregate	Alyans	17.678	7.06	29	0.000

While the γ_d,max_ value of the natural aggregate was 17.678 kN/m^3^, the γ_d,max_ values of the steel slags varied between 15.098 and 21.278 kN/m^3^. The γ_d,max_ value of the slag was generally higher than the natural aggregate, but some values remained low (Table 3). This was because the slags did not have a dense gradation. The w_opt_ values of the steel slags were lower than the natural aggregate (7.06%), except for BOF slags and Yazıcı (EAF slag). Although the water absorption values of the slag samples are higher than the natural aggregate, since steel slags contain coarser particles, they reduce the total surface area and cause a decrease in the w_opt_ values. CBR value of the material to be used as granular fill material should be greater than 25%^22^. Erdemir (24%), Asil (17%), Izmir (10%) and Ozkan (13%) samples were below this limit (Table 3). CBR value of Erdemir sample remained low since it did not show good compaction and had a low γ_d,max_ value (15.098 kN/m^3^). A similar situation was observed in Asil sample, which also had a low γ_d,max_ value (16,687 kN/m^3^). CBR values of Izmir and Ozkan samples were also low due to their high porosity (11.8% and 9.1%, respectively), the low filler content (0.4%) and the coarser particle size (75 mm).

The swelling ratios of all samples were below the limit value (< 1%) except Kardemir sample (1.85%) (Table 2). The main cause of swelling of the slag was the presence of CaO which reacts with water and turns into Ca(OH)_2_. The density of Ca(OH)_2_ is lower than CaO and this reaction causes volume increase^35^. When CaO contacts with water, it is hydrated and can increase its volume by 100%^36^. Permanent settlement (− 0.001%) was observed in Asil sample which had the highest coarse material content (94.62%). The expansion caused by the hydration reaction was tolerated due to the high void ratio of the slag, and settlement caused by the erosion which occurred in the beginning of the hydration reaction by the rigidity loss of CaO in steel slag particles was observed.

**Figure 4:**
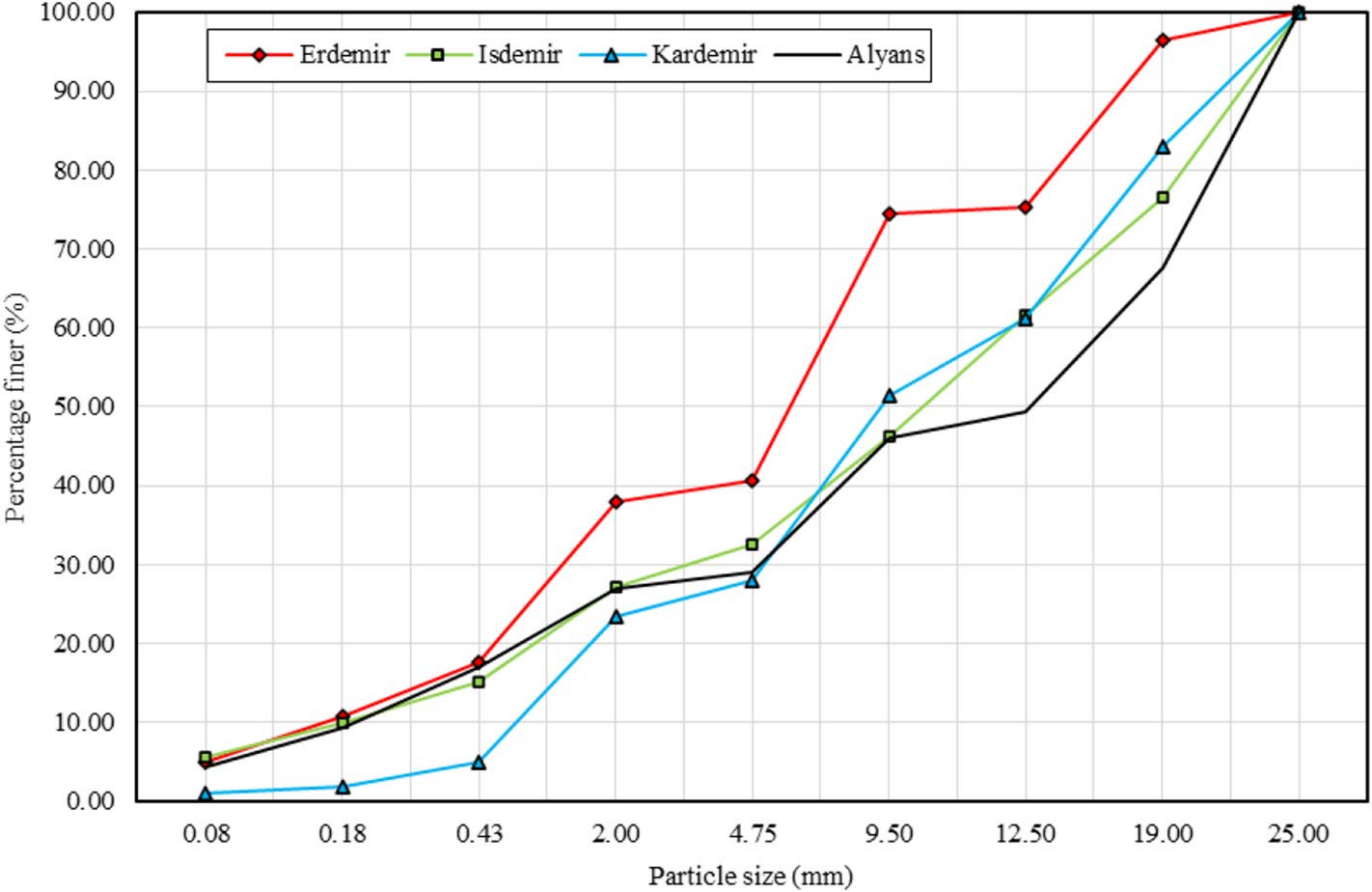
PSD_Dmax25_ curves of BOF slag samples and natural aggregate.

**Figure 5:**
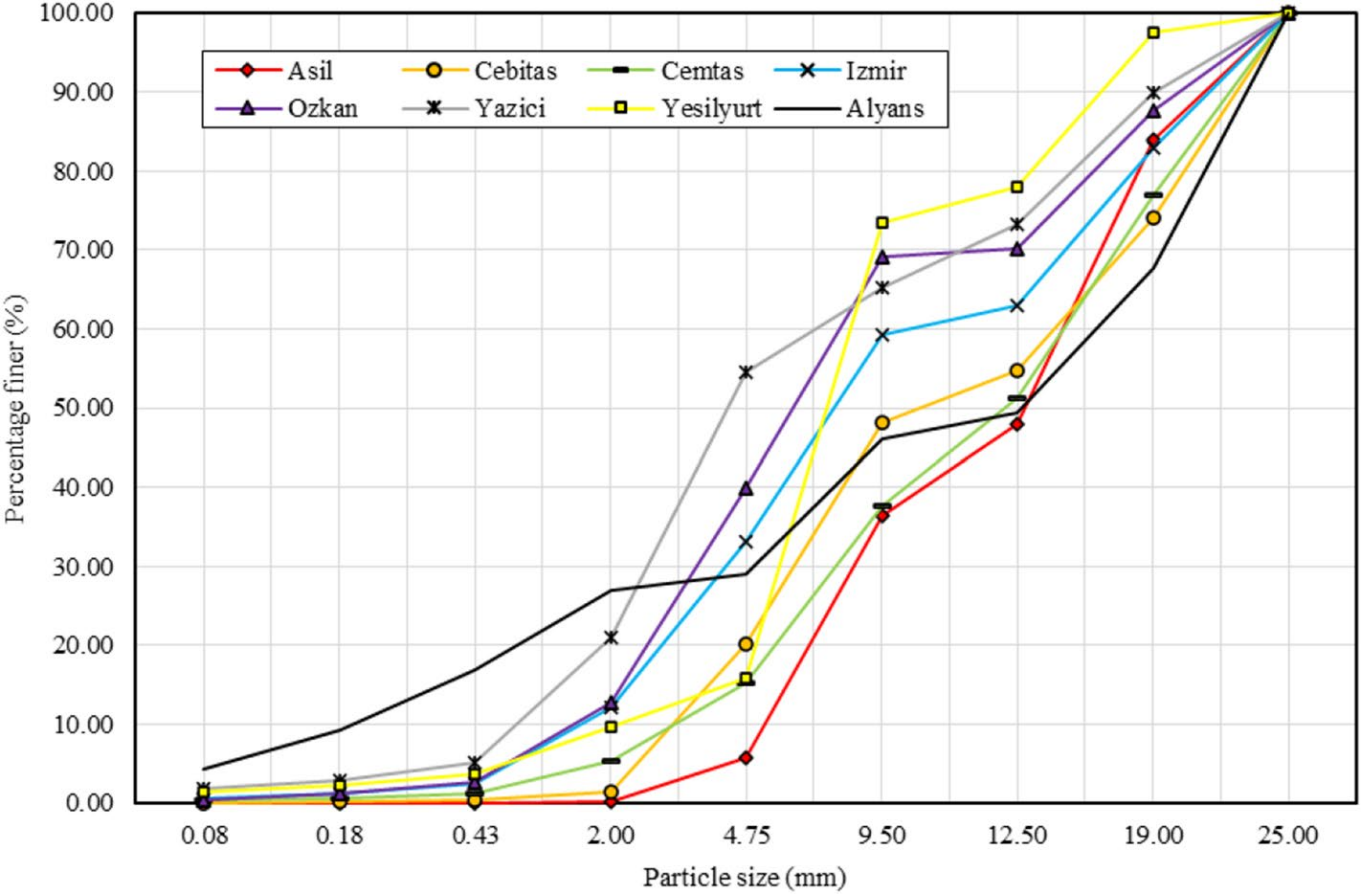
PSD_Dmax25_ curves of EAF slag samples and natural aggregate.

Steel slag has low bearing capacity due to the lack of fine particles (< 4.75 mm) and gradation should be regulated by crushing to increase the bearing capacity and workability^37^. A denser gradation is required to achieve the CBR limit (> 25%) defined in TSSW (2020). For this reason, the maximum particle size of the steel slag was reduced to 25 mm. These materials are named Dmax25 and their PSD are shown in Fig. 4 (BOF) and Fig. 5 (EAF). According to this arrangement, γ_d,max_, w_opt_, CBR (wet) and swelling ratio are given in Table 4.

**Table 4:** Mechanical properties of steel slag samples and natural aggregate after the maximum particle size is reduced to 25 mm.

Slag types	Companies	γ_d,max_ (kN/m^3^)	w_opt_ (%)	CBR, wet (%)	Swelling ratio (%)
BOF slag	Erdemir	20.629	20.89	71	0.955
	Isdemir	20.350	14.49	60	0.770
	Kardemir	19.225	9.77	32	1.228
EAF slag	Asil	19.048	4.15	32	-0.001
	Cebitas	19.082	8.53	78	0.046
	Cemtas	23.079	5.81	42	0.041
	Izmir	21.662	5.09	57	0.032
	Ozkan	20.000	6.98	54	0.033
	Yazici	22.440	10.16	58	0.023
	Yesilyurt	20.524	6.57	43	0.000
Natural aggregate	Alyans	18.948	7.50	38	0.000

After reducing the maximum particle size to 25 mm, the fine material (< 4.75 mm) content of the natural aggregate sample increased to 28.94% (Fig. 4). In the slag samples, the highest ratio of fine material belonged to the Yazici sample (54.58%), and the lowest ratio of fine material belonged to the Asil sample (5.70%) (Fig. 5). The average fine material (< 4.75 mm) ratios of the BOF (Fig. 4) and EAF (Fig. 5) slag samples were 33.71% and 26.39%, respectively. The average fine particle ratio of the BOF slag samples was higher than the natural aggregate (Fig. 4), while the average fine particle ratio of the EAF slag samples was close to the natural aggregate (Fig. 5).

By reducing the maximum particle size to 25 mm, it was seen that the γ_d,max_ values of all steel slag samples were greater than the natural aggregate (18.948 kN/m^3^) (Table 4). In this case, the fine particle ratio of the slags increased and it was observed that the γ_d,max_ values increased as a result of a more dense gradation. Finer size distribution was obtained by reducing the maximum particle size to 25 mm (Figs. 4 and 5). With the increase of the fine particle ratio, the total surface area of the slag increased. Therefore, w_opt_ values increased in all samples except Asil (4.15%) and Izmir (5.09%). Although the maximum particle size was reduced to 25 mm, the Asil sample remained with the lowest fine particle ratio (5.70%) (Fig. 5). This supported that there was no significant increase in the total surface area of the particles in Asil sample. The porous structure is observed more intensely in the coarse particles (≥ 4.75 mm) of the steel slag obtained by rapid cooling and the water absorption ratio in the fine particles is lower than in the coarse particles^13^. This caused no increase in the w_opt_ value of the Asil sample. By reducing the particle size to 25 mm, the fine particle ratio in the Izmir sample increased. Despite the increase in the fine particle ratio, the high-water absorption ratio of the sample and the more porous structure of the coarse particles caused no increase in the w_opt_ value in this sample.

CBR values of all samples increased above the 25% limit value by reducing the maximum particle size to 25 mm (Table 4). Since CBR is a function of gradation, w_opt_ and density^38^, CBR values of all samples increased except Kardemir sample. The decrease in CBR of the Kardemir sample was due to this sample having the highest swelling ratio (1.228%). The fact that the material has a swelling and shrinking structure causes a continuous decrease in CBR, especially in susceptible materials^39^.

**Figure 6:**
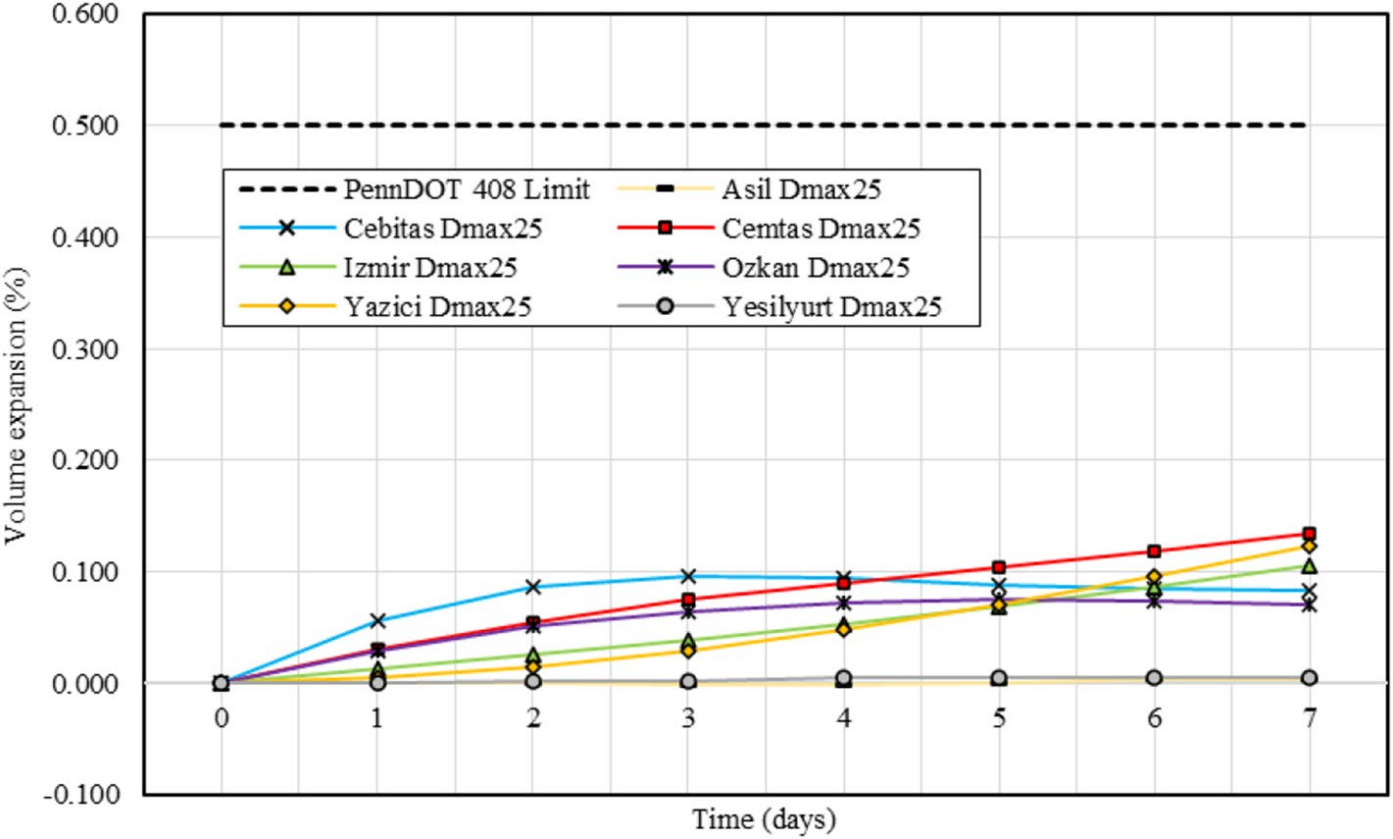
Expansion graphs of EAF slag samples and PennDOT 408 limit.

**Figure 7:**
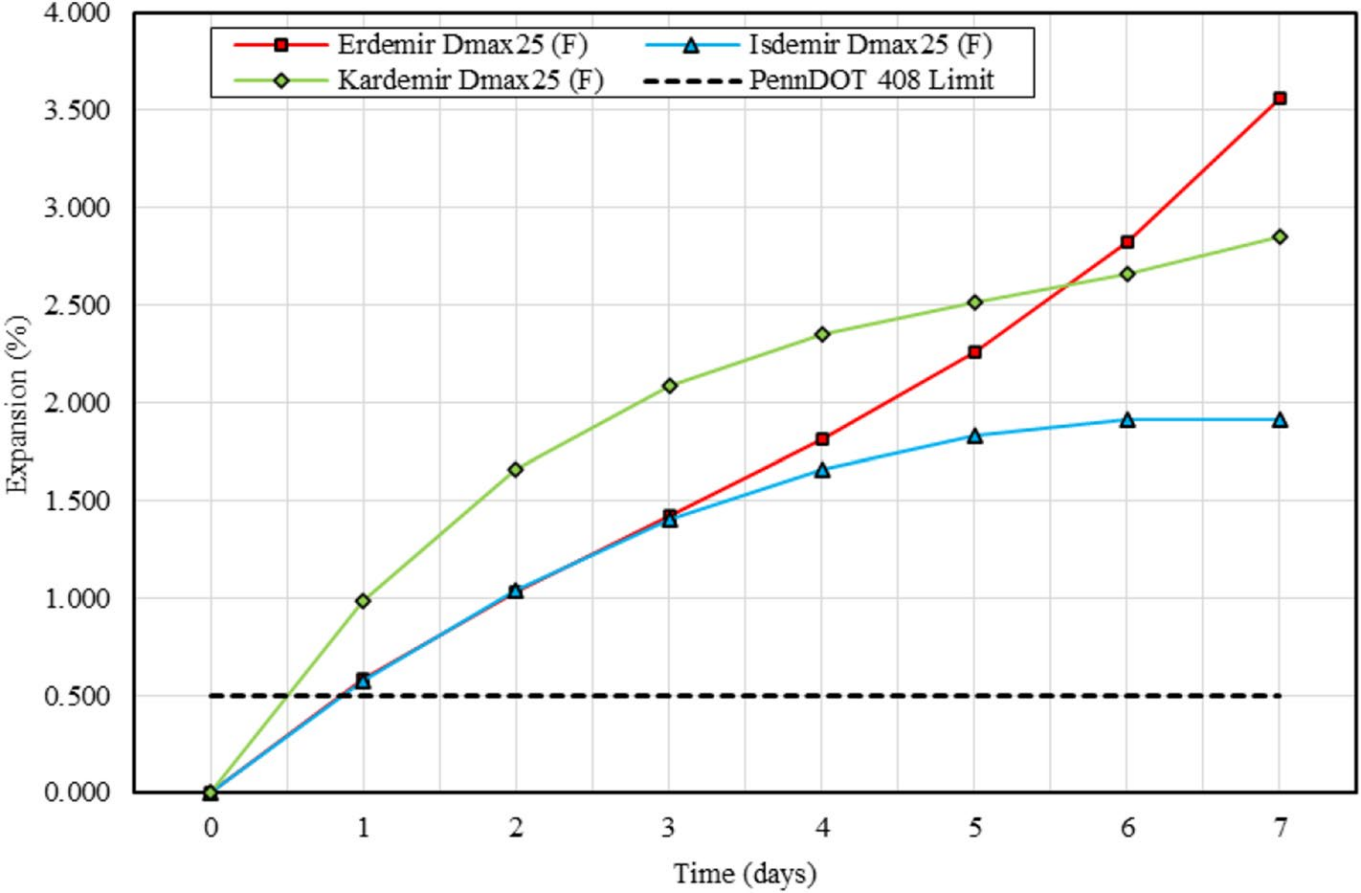
Expansion graphs of BOF slag samples and PennDOT 408 limit.

The most important factor affecting the increase in swelling rate in the sample, which is kept in water for 96 h, is the hydration of CaO. Swelling potentials of steel slags vary depending on the gradation, density and ratio of expandable components. In some cases, the expansion of the components can be tolerated by voids in the slag^35,40^. By reducing the maximum particle size to 25 mm, γ_d,max_ values of all samples increased, except Kardemir sample. When Tables 3 and 4 were compared, it was observed that swelling ratios decreased in Kardemir, Cemtas, Izmir and Ozkan samples. Depending on the decrease in γ_d,max_, swelling rate of the Kardemir sample decreased. Despite the increase in γ_d,max_ in Cemtas, Izmir and Ozkan samples, the decrease in swelling was thought to be due to better interlocking of the particles. Settlement was observed in the Asil sample (− 0.001%) due to the high void ratio and particle erosion due to the highest coarse particle content (Table 4). In Yesilyurt sample, swelling ratio was observed as 0 after 96 h due to the low changes in γ_d,max_, w_opt_ and CBR (Tables 3 and 4). Accelerated expansion tests are conducted and expansion graphs of EAF and BOF slags with maximum particle size reduced to 25 mm (Dmax25) with PennDOT 408 limit (< 0.5%), are shown in Figs. 6 and 7 respectively.

After the expansion tests, even if the expansion values of Cemtas, Yazici and Izmir samples remained below the limit (0.5%) (Fig. 6), an upward expansion trend is observed in these samples. According to these observations the EAF slags should be kept in open air conditions for at least 6 months. After aging, volume expansion rates should be checked again and the slope should be constant or downward for utilization as backfill material in coastal structures.

**Figure 8:**
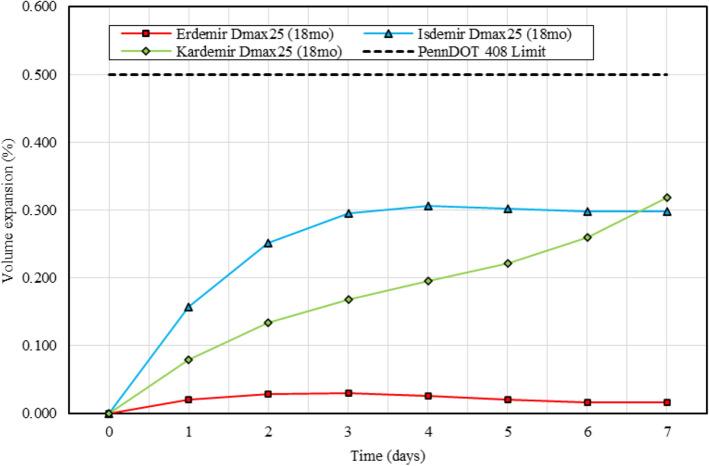
Expansion graphs of BOF slag samples aged for 18 months and PennDOT 408 limit.

BOF slags, Erdemir, Isdemir and Kardemir samples, showed expansion greater than the 0.5% limit defined in PennDOT 408 (Fig. 7). After these three samples were aged in open air for 18 months, the expansion experiments were repeated and the graphs of the expansion rates are shown in Fig. 8.

The expansion rates of the BOF samples fell below the limit value (0.5%) after 18 months of aging (Fig. 8). Since the slope of the Kardemir sample continues upwards, it is recommended to age the BOF slags for 24 months in open air conditions. Afterwards, the expansion rates should be checked again to ensure that the slope is constant or downward.

### Chemical properties of steel slags

Chemical compositions of BOF and EAF slag samples are given in Table 5. Fe_2_O_3_, SiO_2_, Al_2_O_3_ and MgO contents do not have a direct effect on the use of steel slags as backfill materials for coastal structures. However, CaO and MgO are critical for the use of steel slags as they cause an expansion of 98% and 148%, respectively, during their hydration^41^. A maximum free CaO content of 4% was recommended by Shi to keep expansion moderate^42^. For MgO content, the upper limit is defined as 10.5% in the Taiwan South-Star project if it is used as backfill materials for coastal structures^18^. Average free CaO contents of the BOF and EAF slag samples were 7.54% and 0.31%, respectively. CaO, which is considered the primary factor of short-term expansion, is found at a higher rate in BOF slags and this causes BOF slags to expand more. For this reason, BOF slags were aged for 18 months and their free CaO content was measured again. Free CaO contents were determined as 0.46%, 3.03% and 2.26% for Erdemir, Isdemir and Kardemir samples, respectively, after 18 months of aging. Average MgO and Fe_2_O_3_ contents were 3.76% and 15.56% in BOF slags, 3.55% and 25.78% in EAF slags, respectively. It was seen that the average contents of SiO_2_, Al_2_O_3_ and MnO were 12.39%, 3.42% and 3.38% in BOF slags, and 13.30%, 5.73% and 4.47% in EAF slags, respectively.

**Table 5:** Chemical analysis results of BOF and EAF slag samples (wt%).

Slag types	Companies	CaO	Free CaO	MgO	Fe_2_O_3_	SiO_2_	Al_2_O_3_	MnO
BOF slag	Erdemir	33.37	4.69	2.21	17.19	11.86	3.07	2.55
	Isdemir	34.04	6.91	5.56	17.41	10.89	3.58	3.11
	Kardemir	44.84	11.03	3.51	12.07	14.42	3.60	4.48
EAF slag	Asil	25.56	0.34	5.06	17.13	14.30	7.28	4.66
	Cebitas	22.28	0.29	2.59	17.55	14.33	6.10	4.74
	Cemtas	17.70	0.16	5.13	32.26	4.72	2.20	3.88
	Izmir	28.85	0.24	2.78	28.03	19.50	5.86	4.96
	Ozkan	30.07	0.49	4.75	31.49	13.34	4.55	5.64
	Yazici	26.91	0.06	2.65	36.13	11.97	6.70	4.45
	Yesilyurt	34.09	0.56	1.91	17.88	14.96	7.40	2.95

### Environmental assessment

Steel slags are identified with the waste code 10 02 01 (slag processing wastes) and 10 02 02 waste code (untreated slags) in the Annex-4 list (waste from the iron and steel industry) of the Waste Management Regulation and classified as non-hazardous waste^43^. Heavy metal analyzes were carried out to evaluate the environmental effects of steel slags used as backfill materials in coastal structures.

If steel slags are used as backfill materials in coastal structures, heavy metal concentration values should not exceed the values specified in the Water Pollution Control Regulation^44^. When the values in Table 6 are compared with the limit values, it is seen that the slag samples are below the limit values in terms of heavy metal concentrations.

**Table 6:** Heavy metal concentrations of steel slag samples (mg/L).

Slag Types	Companies	Cu	Cd	Cr	Pb	Ni	Zn	Hg	As
BOF slag	Erdemir	< 0.01	< 0.0005	0.019	< 0.0005	0.010	0.04	< 0.0002	< 0.0005
	Isdemir	< 0.01	< 0.0005	0.047	0.005	0.008	0.10	< 0.0002	0.002
	Kardemir	< 0.01	< 0.0005	0.02	0.004	0.005	0.13	< 0.0002	0.001
EAF slag	Asil	< 0.01	< 0.0005	0.07	0.008	0.004	0.34	< 0.0002	< 0.0005
	Cebitas	< 0.01	< 0.0005	0.022	< 0.0005	< 0.001	< 0.015	< 0.0002	< 0.0005
	Cemtas	< 0.01	< 0.0005	0.005	< 0.0005	< 0.001	< 0.015	< 0.0002	0.0005
	Izmir	< 0.01	< 0.0005	< 0.001	< 0.0005	< 0.001	< 0.015	< 0.0002	0.001
	Ozkan	< 0.01	< 0.0005	0.037	< 0.0005	< 0.001	< 0.015	< 0.0002	0.001
	Yazici	< 0.01	< 0.0005	0.001	< 0.0005	< 0.001	< 0.015	< 0.0002	< 0.0005
	Yesilyurt	< 0.01	< 0.0005	0.003	< 0.0005	< 0.001	< 0.015	< 0.0002	< 0.0005
Limit values*	0.01	0.01	0.1	0.1	0.1	0.1	0.004	0.1	

*WPCR^44^.

## Conclusions and recommendations

Large amounts of backfill materials with sufficient strength and durability are required in the backfill of coastal structures. The use of slag, which is generated as a by-product in steel production process, as backfill material in coastal structures is of great importance in terms of protecting the nature, sustainability of natural resources and contributing to the national economy. The soundness loss of steel slags varied between 0.5 and 2.6% and these values were well below the limit value (< 18%). In order for a material to be used as granular fill material, the filler (< 75 µm) ratio is required to be less than 25%. The particle ratio of steel slags smaller than 75 µm varied between 0 and 4.6% and remained well below the limit value. Since the steel slags have non-plastic properties and are little affected by freezing and thawing, it has been evaluated that there will not be a negative situation in case of contact with precipitation or sea water.

It was determined that the density, porosity, water absorption and LA abrasion loss of steel slags were generally higher than natural aggregate. High density is caused by metal oxides in the slag and this may increase the transportation costs of the steel slag. However, due to the fact that steel plants are generally located in port areas, there may be short transportation distances for coastal port structures to be built in the same region. Due to its porous structure, the coastal backfill to be constructed with slag will have high permeability and will contribute to the easy drainage of the precipitation water coming from the surface. In addition, the low capillary effect will prevent the rise of the water coming from the base. In order to reach CBR limit (> 25%), the maximum particle size of the steel slags was reduced to 25 mm within the limits. In this particle size, it was observed that CBR values of the slag samples generally gave better results compared to the natural aggregate, except for Kardemir and Asil samples. In accelerated expansion test despite the low expansion of EAF slags, the upward expansion trend of the Cemtas, Yazici and Izmir samples continued. For this reason, it has been suggested that the EAF slag should be aged for at least 6 months in open air conditions before being used as backfill material and the expansion test should be repeated at the end of aging. It was observed that BOF slags exceeded the limit value and after the samples were aged for 18 months, the expansion values of all samples remained below the limit, but the expansion trend line of the Kardemir sample continued to move upward. Accordingly, it has been suggested that BOF slags should be aged for at least 24 months in open air conditions before being used as backfill material and the expansion test should be repeated at the end of aging. In addition, heavy metal concentrations were analyzed to evaluate the environmental effects of using these slags as backfill material in coastal structures. The findings show that steel slags can be successfully used as backfill material in coastal structures without any negative effect on the environment.

## Data Availability

The data that support the findings of this study are available from the corresponding author upon request.
